# Antimicrobial Potential of *Streptomyces* spp. Isolated from the Rift Valley Regions of Ethiopia

**DOI:** 10.1155/2022/1724906

**Published:** 2022-06-13

**Authors:** Firew Elias, Sudhamani Muddada, Diriba Muleta, Belachew Tefera

**Affiliations:** ^1^Department of Biotechnology, Koneru Lakshmaiah Education Foundation, Vaddesswaram, AP, India; ^2^Animal Products Veterinary Drugs and Feed Quality Control Centre, Addis Ababa, Ethiopia; ^3^Environmental Biotechnology Unit, Institute of Biotechnology, Addis Ababa University, Addis Ababa, Ethiopia

## Abstract

The study was undertaken to isolate, screen, and identify actinomycetes with antimicrobial metabolites. Twenty-one composite soil samples were randomly collected from various unique agroecological niches in the Rift Valley of Ethiopia. The soil samples were serially diluted and spread on starch casein agar medium supplemented with 50 *μ*g/ml cycloheximide and 25 *μ*g/ml nalidixic acid. Two hundred and forty-nine (249) actinomycetes cultures were isolated and screened by cross streaking against various human pathogens. Twenty-four isolates with pronounced antimicrobial activity were selected for identification and further screening. Among the isolates, 172 (69.1%) showed antimicrobial activities against tested pathogens. The inhibition zone of the isolates ranged from 5 ± 0.31 to >40 mm during primary screening. The antimicrobial activity of the crude extracts of promising isolates showed a statistically significant difference (*P* < 0.05) between them and the control. The isolates RVE129 and RVE217 showed the maximum zone of inhibition at 27 ± 0.6 mm and 26 ± 0.6 mm, respectively, against *S*. *aureus*, and the results were higher than the standard drug streptomycin (25 ± 0.58 mm). The inhibition zone of crude extracts from RVE129 was at the maximum of 22 ± 0.0 mm against *P*. *aeruginosa*, almost comparable to the standard drug streptomycin (24 ± 0.58 mm). Crude extract from the isolates RVE129 and RVE187 showed higher inhibition zones of 22 ± 0.6 mm and 16 ± 0.33 mm against *A*. *niger* ATCC10535, which, however, were smaller than those obtained with the standard drug amphotericin B (29 ± 0.6 mm). Twenty-four actinomycete strains with remarkable bioactivity were characterized using various cultural, morphological, physiological, and biochemical characteristics and assigned under the genus *Streptomycetes*. The finding of the current study indicates that *Streptomyces* sp. isolated from the Rift Valley of Ethiopia was found to possess a broad spectrum of bioactivity against a range of human pathogens.

## 1. Introduction

One of the greatest achievements in the previous seven decades was the discovery of a novel antimicrobial agent to battle life-threatening infectious diseases caused by microbes [1]. However, enduring infectious diseases, rapidly emerging drugs, and multidrug resistance by pathogenic microorganisms compromise the efficacy of existing antibiotics. This triggered the need for the screening of microorganisms from natural sources for new antibiotics with novel biomolecules that are active against a wide range of pathogens [2, 3]. Natural products are widely recognized as one of the most important sources of bioactive secondary metabolites with novel structural and functional diversity for the treatment and prevention of human diseases [3]. Among microbial natural sources, actinomycetes are a potential source of a great number of economically and industrially important bioactive secondary metabolites many of which are diverse chemical structures and potent biological activities [4, 5].

Actinomycetes are diverse phyla of ubiquitous Gram-positive, aerobic, slow-growing bacteria that usually grow with characteristic filamentous and sporulating morphology with >55% guanine and cytosine in their DNA [6]. Of all the reported bioactive compounds of microbial origin, approximately 42% have been presently obtained from actinomycetes [7]. Currently, the majority of the commercially and medically known antibiotics have originated from a different genus of actinomycetes [6, 7]. Actinomycetes have also been the source of many industrially and clinically important bioactive metabolites in treating cancer, inflammatory diseases, allergies, anthelmintics, autoimmune diseases, infections, and others [8, 9]. Among actinomycetes genera, *Streptomyces* are the largest genus and the most frequent producer of antibiotics which is continuously being explored for antimicrobial drug discovery [9, 10].

Actinomycetes have been isolated from a wide range of habitats: soil, water, air, forest manure, river mud, and compost, and also in extreme environments such as mangroves, marine, deserts, hot springs, and caves as well as being associated with various plants and animals [10–12]. Although actinomycetes have been widely distributed in nature, soil, in particular, is the largest reservoir of actinomycetes that has been producing the most useful bioactive drugs to treat a wide range of human and animal pathogens [13]. Although soils have been screened to produce innovative bioactive compounds with many agricultural, pharmaceutical, and industrial applications, only a small portion of the earth's surface as well as actinomycetes taxa has been sampled and studied [13, 14]. The major challenge in the traditional approach of screening antibiotics is the isolation of common microorganisms capable of producing known bioactive metabolites from conventional environments, which have already been extensively examined [14]. Several researchers have focused to screen novel and biotechnologically exploitable microorganisms that may yield new and diverse antimicrobial activities from previously unstudied and diverse geographical regions which could have evolved differently from those that had already been explored [12–15].

Therefore, current isolation and exploitation strategies for isolation of actinomycetes producing novel bioactive metabolites require new sample sources from uncommon and unexplored habitats [16]. In this respect, the current study was designed to explore actinomycetes possessing antibacterial and antifungal activities from soil samples collected from largely unexplored and various ecological niches in the Rift Valley of Ethiopia.

## 2. Methodology

### 2.1. Sample Site and Sample Processing

Samples of soil were collected from seven diverse geographical regions in the Rift Valley of Ethiopia, namely, Debre Zeit, Adama, Awash, Ziway, Arsi Negele, Hawassa, and Arba Minch. Sample collection sites encompassed diverse ecological habitats including uncultivated soil, river/lake sediments, and rhizosphere soil of plants. From each site, five plots were prepared randomly and a total of 105 (7 × 3 × 5) soil samples were taken using a sterile spatula and sterile glove. To obtain one representative sample per plot, the collected soils were mixed to make 21 (3 × 7) composite soil samples per sample source and sampling site. Soil samples were kept in sterile sealed polythene bags, placed in an icebox [17–19], and transported to the Microbiology Laboratory at Animal Products Veterinary Drugs and Feed Quality Control Center, Addis Ababa, Ethiopia.

### 2.2. Isolation and Maintenance of Isolates

The composite soil samples were ground with mortar and pestle and sieved through a 250 *μ*m mesh size sieve and air-dried in the laminar flow (LAF) at room temperature overnight [20]. According to Salaria and Furhan [21], actinomycetes were isolated from soil samples on starch casein agar medium supplemented aseptically with cycloheximide (50 *μ*g/ml) and nalidixic acid (25 *μ*g/ml) to reduce microbial contamination. One gram of dried composite soil sample was dissolved in 9 ml of 0.85% normal saline solution, and 10-fold serial dilutions were prepared aseptically in the test tube (10^−1^ to 10^−7^). Aliquots of 100 *μ*l from each sample of 10^−4^ to 10^−5^ dilutions were transferred and spread aseptically on starch casein agar (SCA) in triplicate. Finally, pure isolates were individually transferred into SCA slants and temporarily kept at 4°C after incubation at 30°C for 7–15 days.

### 2.3. Test Pathogens

A total of eight test organisms were used for antimicrobial screening of antibiotic-producing actinomycetes. The test organisms were *Staphylococcus aureus* ATCC-259233, *Staphylococcus epidermidis* ATCC-12228, *Salmonella typhi* ATCC-13311, *Pseudomonas aeruginosa* ATCC-27853, *Klebsiella pneumonia* ATCC-700603, *Escherichia coli* ATCC-25922, *Salmonella typhi* ATCC-14028, and *Aspergillus niger* ATCC-10535. The Microbiology Laboratory, Traditional and Modern Medicine Research Directorate, Ethiopian Health and Nutrition Research Institute, provided all standard bacterial and fungal strains (EHNRI).

### 2.4. Inoculum Preparation and Standardization

The inoculum was prepared and standardized to the density of the screening test done by the method described in [22]. A loopful colonies of test pathogens from fresh culture were transferred to test tubes containing sterile saline (0.85%) and used to match turbidity equivalent to 0.5 McFarland standards which are equivalent to a cell density of 10^6^–10^8^ CFU/mL for bacteria, whereas 2.5 × 10^3^ CFU/mL for fungi was used. Then, the optical densities of the suspensions were fitted to 0.5 McFarland standards at 530 nm (OD530) in a spectrophotometer (JANEWAY, London).

### 2.5. Preliminary Screening for Antimicrobial Analyses

Two hundred and forty-nine (249) actinomycete isolates with the antagonistic potential against bacterial pathogens were preliminarily screened by the cross-streak assay, and antifungal activities were tested by the spot inoculation method [23]. Then, they were primarily screened for antimicrobial activity using Mueller–Hinton Agar (MHA) medium for bacteria and slightly modified potato dextrose agar supplemented with (g/L: 0.5 K_2_HPO_2_, 0.75 CaCO_3_, 0.5 MgSO_4_.7H_2_O, and 2 NaCl) for fungi. Each 3-day-old actinomycete culture was streaked as a straight single line in the middle of MHA plates and incubated at 30°C for 3 to 4 days. After observing the growth of actinomycete isolates, a fresh culture of each bacterial pathogen (0.5 McFarland standards) was streaked at a right angle to the streak of the isolates but not in contact with the colony. Then, the bioactivity of isolates against fungi was tested on plates with modified potato dextrose agar inoculated with 3-day-old actinomycetes cultured in two sides about 30 mm from the middle of the plates and incubated at 24°C for 4 days and overnight at 37°C for bacteria. The inhibition zones were observed, and the results were recorded in millimeters.

### 2.6. Secondary Screening

#### 2.6.1. Extraction of Crude Antimicrobial Compounds

Solvent extraction of the bioactive metabolite from the selected actinomycetes isolates which exhibit a broad-spectrum activity in primary screening was done by liquid-state fermentation as described in [22,23] with some modifications. Briefly, two milliliters from each fresh culture were transferred into 100 mL of (ISP-2 broth) yeast extract malt extract dextrose broth in conical flasks under sterile conditions and were placed into a shaking incubator at 28 ± 2°C for 8 days. After fermentation, each isolate's supernatant from the broth medium was centrifuged for 10 min at 10,000 rpm. The fermented broth culture containing bioactive metabolite was separated from the solid residue through Whatman No. 1 filter paper. The supernatant broth was collected and mixed 1:1 (*v*/*v*) with an equal amount of ethyl acetate (50 mL). The bioactive compound-containing organic solvent phase was then separated from the aqueous phase in a separatory funnel, collected, and evaporated in a vacuum rotary evaporator at 100 rev/min and 60°C [23]. The completely dried residues from each isolate were weighted separately using a balance and dissolved in dimethyl sulfoxide (DMSO) and placed in small vials at 4°C to determine the antimicrobial activity and further analysis.

#### 2.6.2. Disc Diffusion Assay

The antagonistic activities of the crude extract of each isolate were tested by disc diffusion assay using the protocol described in [19]. Briefly, inoculum prepared from each bacterial and fungal suspension (1 mL) was mixed with 9 mL of sterile Mueller–Hinton broth (MHB) and compared the turbidity with standard 0.5 McFarland solutions. A sterilized cotton swab dipped into the overnight culture of each test bacterial pathogens and fungal culture (*Aspergillus niger* ATCC-10535) were swabbed separately on the entire sterile Muller–Hinton Agar (MHA) plate for bacteria and Sabouraud Dextrose Agar (SDA) plate for fungi. Fifty *μ*l crude extract of each actinomycete isolate supernatant, streptomycin (25°*μ*g/ml) for bacteria and amphotericin B 50 *μ*g/ml for fungi as positive controls, and DMSO solvent (as negative controls) was loaded into each sterilized 6 mm diameter Whatman No. 1 paper discs kept on the inoculated agar plates and placed in the refrigerator for 2 hrs. The plates were then incubated for 24 hrs at 37°C for bacteria and 48 hrs at 28°C for fungus, and the diameter of the inhibitory zone surrounding each disc was measured and the findings reported in millimeters.

### 2.7. Identification of Selected Actinomycete Isolates

Twenty-four potential pure isolates of the actinomycetes were identified based on cultural, physiological, and biochemical characterizations as per the standard protocol suggested by Bergey's Manual of Determinative Bacteriology and the International Streptomyces Project (ISP) [24].

#### 2.7.1. Morphological Characterization

Morphological characteristics of the isolates were determined as per the guidelines of international streptomyces project [24]. The growth pattern, color of aerial mycelium, substrate mycelium, and diffusible pigments of isolated actinomycetes were all examined. Pure isolates were streak plated onto various media (ISP 2–7 and starch casein agar) and cultured for 7–14 days at 30°C. Cover slip culture and Gram staining procedures were used to examine macromorphology. The morphology of spore bearing hyphae and the spore chain were determined by the coverslip method using a well-grown sporulated culture plate in starch casein agar plate at a 45° inclination using a light microscope (BX-51; Olympus, Japan). It was incubated for four days at 30 C, after which the cover slip was removed with sterile forceps and placed on a separate clean glass slide, which was then viewed using an oil immersion objective.

#### 2.7.2. Biochemical and Physiological Characterization

A loopful of pure test isolate culture from 7 days of incubation was poured into starch casein broth and cultured for 4 days at 30 C. After growth, about 0.1 ml of the culture suspension was used for biochemical and physiological tests following the methods in [25, 26]. Physiological characteristics such as temperature tolerance, NaCl tolerance, and pH level were investigated. Various enzymatic activity tests like catalase, oxidase, nitrate reductase, amylase, caseinase, gelatin hydrolysis citrate utilization, and melanin production tests were also performed according to the previous description in [9, 18].

### 2.8. Statistical Analysis

To compare the significant level of bioactivity between the potent isolates and the positive control, the antimicrobial activity data from the secondary screening were analyzed using one-way ANOVA in Statistical Package for Social Sciences (SPSS) version 20. The results were accepted to be statistically significant with *P* ≤ 0.5.

## 3. Results

### 3.1. Isolation of Actinomycetes

**Figure 1:**
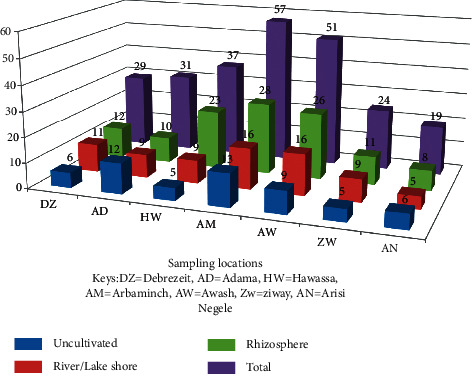
Number of actinomycete isolates from the Rift Valley areas of Ethiopia.

A total of two hundred and forty-nine different actinomycetes were isolated from twenty-one composite soil samples collected from diverse agroecological habitats, namely, river/lake sediments and uncultivated and rhizosphere soils of seven studied areas (Debre Zeit, Adama, Awash, Ziway, Arsi Negele, Hawassa, and Arba Minch) of the Rift Valley of Ethiopia. The total number of actinomycetes isolated from each of the study areas with its specific sample source and sampling site is demonstrated in Figure 1.

Out of the isolated actinomycetes, 29 (11.65%) were isolated from Debre Zeit, 33 (14.06%) from Adama, 57 (21.29%) from Arba Minch, 24 (9.64%) from Ziway, 19 (7.63%) from Arsi Negele, 37 (14.89%) from Hawassa, and 51 (20.88%) from Awash (Figure 1). Of the isolates, the highest numbers of actinomycetes, 108, which account for 43.37%, were isolated from rhizosphere soil of different plants, and the least, 56, which account for 22.49%, were obtained from uncultivated land (Figure 1). Furthermore, the potent isolates in terms of activity spectra against test organisms, namely, the isolates RVE129 and RVE187, were obtained from the rhizosphere soil of Hawassa and Awash in the Rift Valley area of Ethiopia, respectively.

### 3.2. Screening of Isolated Actinomycetes for Antimicrobial Potential

#### 3.2.1. Preliminary Antimicrobial Assay

All 249 representative actinomycete isolates were primarily screened on agar medium against eight test organisms to determine their antagonistic potential by the cross-streak method. Of the total isolates, 172 (69.1%) had antimicrobial activity, of which 144 (83.72%) isolates showed antibacterial activity, 30 (17.45%) isolates showed antifungal activity against *A*. *niger*, and 51 (29.65%) isolates showed both antibacterial and antifungal activity (Table 1).

However, 77 isolates (30.92%) showed no antibacterial activity against any of the pathogens. Out of the isolated active actinomycetes, samples from Awash gave the largest number of 51 (35.42%) actinomycete colonies with antimicrobial activity (Table 1). On the other hand, the least number of active actinomycetes were isolated from Ziway 9 (5.23%).

**Figure 2:**
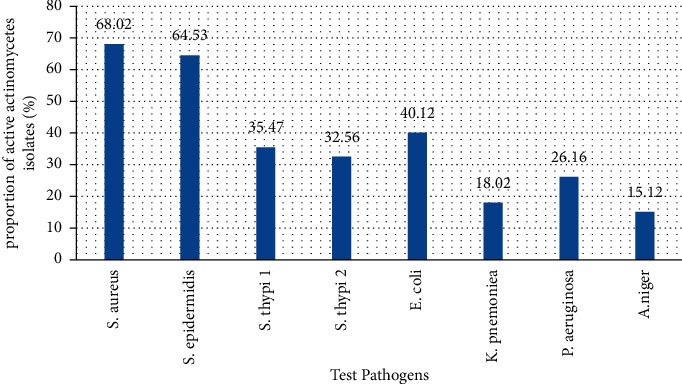
Proportion of active actinomycetes against different test pathogens.

**Figure 3:**
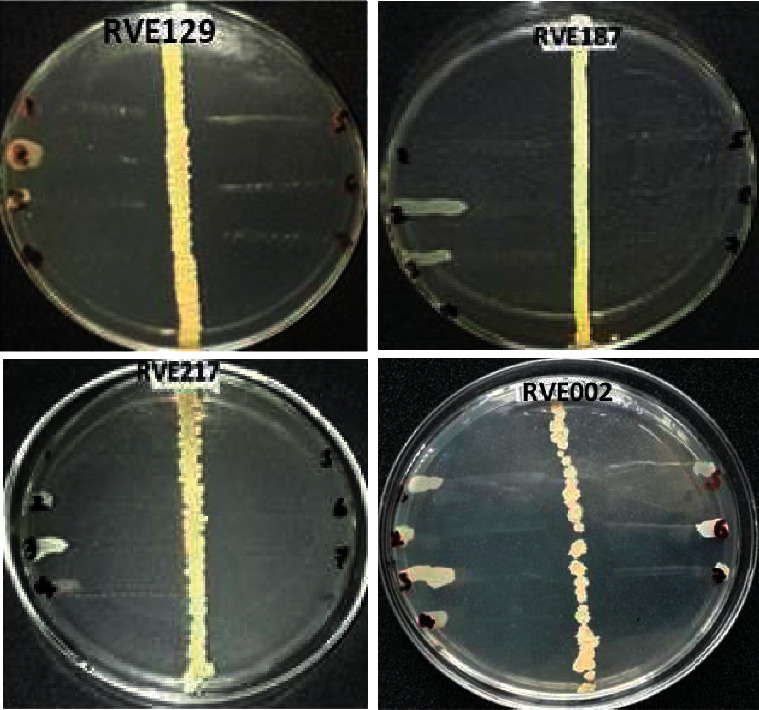
The inhibition zone of selected isolates against tested pathogens. The vertical streak is the actinomycete strains to be tested and the perpendicular streaks with numerical digits are the test organism *Salmonella typhi* ATCC-13311 (1), *Pseudomonas aeruginosa* ATCC-27853 (2), *Klebsiella pneumoniae* ATCC-700603 (3), *Escherichia coli* ATCC-25922 (4), *Salmonella typhi* ATCC-14028 (5), *Staphylococcus aureus* ATCC-259233 (6), and *Staphylococcus epidermidis* ATCC-12228 (7).

Amongst the active actinomycete isolates screened for antimicrobial activities, the highest, which accounted for 117 (68.02%) of the isolates, displayed antagonistic activity against *S. aureus* ATCC29213. The least inhibited organisms were *P. aeruginosa* ATCC27853 and *K. pneumonia* ATCC-25922, which were inhibited by 45 (26.16%) and 31 (18.02%) isolates, respectively (Figure 2). The bioactivity of selected isolates against test pathogens in primary screening is summarized in Figure 3. Interestingly, the isolates RVE002, RVE129, RVE187, and RVE217 exhibited the best antagonistic activity against the tested organisms, exhibiting complete inhibition of the growth of some of the test pathogens (Table 2 and Figure 3).

#### 3.2.2. Secondary Screening

The ethyl acetate crude extracts of twenty-four active actinomycete isolates showed antimicrobial activity against the tested pathogens with inhibition zones ranging from 7 ± 0.6 mm to 27 mm (Table 3). The detailed results of secondary screening for bioactivity of active actinomycetes are shown in Table 3.

The bioactivity testing of the ethyl acetate extract of potent actinomycete isolates was shown to be significantly different (*P* < 0.05) between them compared to the standard drug (Table 3). The isolates of RVE129 and RVE217 showed the maximum zone of inhibition at 27 ± 0.6 mm and 26 ± 0.6 mm, respectively, against *S*. *aureus*, and the results were higher than the standard drug streptomycin (25 ± 0.58 mm). The inhibition zones of crude extracts from RVE187 and RVE217 were at a maximum of 25 ± 0.6 mm and 25 ± 0.0 mm, respectively, against *S*. *epidermidis*, which was comparatively similar to the positive control drug (25 ± 0.8 mm). The isolate RVE129 tested against *P*. *aeruginosa* showed good bioactivity (22 ± 0.0 mm) compared to the standard drug streptomycin (24 ± 0.58 mm) (Table 3). A significant (*P* < 0.05) variation of antifungal activity was recorded among the ethyl acetate extract from selected actinomycete isolates. The isolate RVE129 (22 ± 0.6 mm) inhibited *A*. *niger* better than the other isolates but less than the control standard drug amphotericin B 50 *μ*g/ml (29 ± 0.6 mm).

### 3.3. Characterization of Selected Actinomycete Isolates

#### 3.3.1. Morphological and Cultural Characterization

The actinomycetes isolated from soil samples of the Rift Valley of Ethiopia are all Gram-positive filamentous bacteria. The promising isolates exhibited growth variations among the tested media. However, most of the isolates exhibited abundant to moderate growth on yeast extract malt extract dextrose agar (ISP-2), starch-inorganic salts agar (ISP-4), peptone yeast extract agar (ISP-6), and starch casein agar medium (Table 4).

#### 3.3.2. Physiological and Biochemical Characteristics

The growth characteristics of the selected isolates were examined at different incubation temperatures, pH levels, and salt concentrations. The physiological and biochemical characteristics of the 24 selected isolates are summarized in Table 4. The results showed that most isolates could grow well at pH levels ranging from 4 to 11, with pH 7.5 being the optimum. The temperature range for the isolate growth was determined to be between 20 and 45°C, with 25°C and 30°C being the optimal temperatures. The isolates were capable of growing at sodium levels up to 7.5%, with optimum growth being observed in media supplemented with 2.5% and 5% NaCl (*w*/*v*). Most isolates were positive for chitinase, oxidase, and catalase tests and showed a negative result for indole production. All potent isolates in this study had shown positive results for starch hydrolysis, but they demonstrated some differences against reducing nitrate salts and in the decomposition of urea. Except the *Streptomyces* strains RVE029, RVE116, and RVE 248, the rest strains were found to be positive for melanin production. Out of the 24 tested isolates, 20 (83.3%) could hydrolyze casein, except for the actinomycete isolates RVE125, RVE172, RVE217, and RVE247. Most isolates (20, 83.33%) showed the positive utilization of citrate as the main carbon source, and 16 (66.7%) of the isolates were able to hydrolyze gelatin (Table 4).

## 4. Discussion

Currently, the threat of drug-resistant organisms to existing antibiotics has become a challenge across the world, predominantly in developing countries including Africa. Therefore, it is an urgent need for searching new, potent, and broad-spectrum antimicrobial agents against infectious pathogens from microbial sources [7]. Actinomycetes especially *Streptomyces* have proven potential to produce bioactive compounds and are the richest sources of secondary metabolites including novel antibiotics [8–10]. Thus, exploration of bioactive metabolite compounds for potential use in biotechnological, agricultural, and biopharmaceutical applications from actinomycetes isolated from unexplored and extreme environments has required considerable attention. Recently, several researchers reported the potential of the most extreme and unstudied environments such as marine water, deserts, volcanic areas, and mangrove forests as new sources of promising antimicrobial compound producing actinomycetes [4, 7, 12, 14–16, 27]. Taking this rationale into account, the current work focused on the isolation of bioactive actinomycetes and screened for potent activity against antibacterial and antifungal activity from the previously unstudied, diversified geographical regions of the Rift Valley of Ethiopia. The highest numbers of actinomycetes were isolated from the rhizosphere soil of different plants in the current investigation. This demonstrated that soil samples collected from various biological environments and ecological habitats of the Rift Valley of Ethiopia support a diverse number of actinomycetes, but there is a higher occurrence of actinomycetes in the rhizosphere soil of different plants than in other soils. Sharma and Thakur [12] confirmed this finding, indicating that samples from rhizosphere soil included higher percentages of actinomycetes. The current findings are also consistent with [18], which indicated that rhizosphere soils had the highest number of actinomycetes. Rhizosphere soils were effective sources for isolating actinomycetes with potential antimicrobial bioactive substances [19]. The effectiveness of actinomycetes in the rhizosphere region could be attributed to the availability of plant nutrients and other exudates. Furthermore, the most potent isolates in terms of activity spectra against test organisms, namely, the isolates RVE129 and RVE187, were obtained from the rhizosphere soils of Hawassa and Awash in the Rift Valley area of Ethiopia, respectively. Of the total isolates, 172 (69.1%) showed varying levels of antagonistic activity against tested bacteria and fungi (*A*. *niger* ATCC10535). This indicated that the occurrence of actinomycete isolates with antimicrobial activity in the current research region was higher than 45.3% [8] and lower than 71.9% [12]. These relatively high proportions of numbers of isolates from the study area suggest that samples of the Rift Valley of Ethiopia have a high potential for the isolation of actinomycetes having antimicrobial activity.

The present study showed, during the primary screening, the maximum antibacterial activity against *S*. *aureus* (>40 mm), *S*. *epidermidis* (>40 mm), *E. coli* (>33 mm), *S. typhi* (>35 mm), *K*. *pneumoniae* (30 ± 0.20 mm), *P*. *aeruginosa* (32 ± 0.58 mm), and *A*. *niger* (20 ± 0.0 mm) was greater than the previous findings. Sapkota et al. [13] observed that the strongest antagonistic activity was 19 mm against *E*. *coli* (ATCC 25922), 30 mm against *S*. *aureus* (ATCC 25923), and 18 mm against *K*. *pneumonia* (ATCC 700603). However, none of the isolates showed antibacterial activity against *P*. *aeruginosa* (ATCC 27853). According to Kumari et al. [18], the maximum zone of inhibition was 33 mm against *S*. *aureus*, 33 mm against *E*. *coli*, and 30 mm and 8 mm against *A*. *niger*. However, none of the isolates showed antibacterial activity against *P*. *aeruginosa* (ATCC 27853). The antagonistic activity of actinomycetes strains showed different degree of bioactivity profiles that might be due to the difference in the bioactive secondary metabolite that were produced by isolates. This is similar to the trends demonstrated by Sharma and Thakur [12] who suggest that the different natural environments from which the isolates recovered influences their secondary metabolite biosynthesis potential.

In the current study, the activity of ethyl acetate extract of crude antimicrobial compounds was compared with that of control antibiotics (streptomycin). Although control antibiotics had a greater inhibition zone than the crude ethyl acetate extracts of actinomycetes in most cases, the isolates RVE129 and RVE217 showed the greatest zone of inhibition against *S*. *aureus*, as well as the isolate RVE187, tested against *K*. *pneumoniae*, had a zone of inhibition comparable to positive control. All the isolates had good antifungal activity against *A*. *niger*. However, none of the isolates had a higher inhibition zone than amphotericin B (positive control). This could be due to impurities in the extract or impurities in the extract obstructing extract diffusion in the agar plates, lowering the bioactivity of the extracts. The metabolites from these isolates could be purified further to get their effectiveness and composition to compare with a pure antibiotic drug that was already in clinical use, as reported earlier in [18, 22].

The promising isolates exhibited growth variations among tested media. However, good growth of the isolates was exhibited on yeast extract malt extract dextrose agar (ISP-2), starch-inorganic salts agar (ISP-4), peptone yeast extract agar (ISP-6), and starch casein agar medium, and this may be due to the sufficient amount of nutrient composition available in the media and the nutritional adaptability of the isolates. The optimum temperature for the growth of potential actinomycetes isolates was found at 25 and 30°C, they could be placed in the mesophilic range. The isolates were also found to grow with optimum pH 7–8 and with optimum growth at 2.5–5% NaCl concentration. From these observations, it was clear that isolates from the Rift Valley of Ethiopia could be placed in moderately halo-tolerant. In comparison to the present findings, Ameerah and co-workers [27] reported growth of actinomycetes in up to 10% NaCl concentration (optimum at 5% *w*/*v*), pH range of growth from 5 to 9 (optimum at pH 8.0), and growth in temperature range 25–40°C (30°C). In this study, most isolates were positive for amylase, caseinase, chitinase, oxidase, and catalase tests. Most isolates also showed the positive utilization of citrate as the main carbon source. Melanin production was also seen in almost all of the studied strains, which is a key descriptive characteristic of *Streptomyces* sp. [23]. A similar study was carried out by Sapkota et al. [13], in which 78% of the total *Streptomyces* could exhibit demonstrate one or more enzymatic activities. This indicates that actinomycete isolates possess the potential to produce a broad range of enzymes, which may be the mechanism for survival in a competing environment. These isolates were categorized under genus *Streptomyces* based on cultural, morphological, biochemical, and physiological characteristics. The results can also be in-line with earlier studies who have documented similar characteristics description of the genus *Streptomyces* isolated from soil [7, 13, 18]. Furthermore, our findings matched the *Streptomyces* genus description in Bergey's Manual of Determinative Bacteriology, indicating that these isolates were found to be compatible with those of the *Streptomyces* genus [24].

## 5. Conclusion

In conclusion, this study provides a proof that the diverse ecological habitats in the Rift Valley of Ethiopia are a reservoir of potentially active actinomycetes. Our findings also show that *Streptomyces* sp. isolated from the research area has a broad spectrum of bioactivity against a wide range of human pathogens, including drug-resistant *Staphylococcus aureus*. In addition, the antimicrobial compounds obtained from *Streptomyces* strains RVE002, RVE129, RVE187, and RVE217 are comparatively similar or higher in some cases to standard drug; these would be helpful for large-scale manufacturing of antibiotic drug against a wide range of pathogens. The present investigation suggests the need for further characterization and purification of the bioactive molecules obtained from the potent isolates.

## Figures and Tables

**Table 1:** Proportion of antimicrobial activity of actinomycete isolates.

Sample locations	Total no. of active isolates	No. of active isolates against bacteria	Proportion of active isolates AB (%)	No. of active isolates against fungi	Proportion of active isolates AF (%)	No. of active isolates against bacteria + fungi	Proportion of active isolates AF + AB (%)
Adama	19	17	9.88	2	1.16	7	4.07
Awash	51	40	23.26	11	6.40	9	5.23
Ziway	9	8	4.65	1	0.58	7	4.07
Arsi Negele	19	12	6.98	7	4.07	3	1.74
Hawassa	26	22	12.79	4	2.33	5	2.91
Arba Minch	35	31	18.02	4	2.33	14	8.14
Debre Zeit	15	14	8.14	1	0.58	6	3.49
Total	172	144	83.72	30	17.45	51	29.65

Note: AF: antifungal activity; AB: antibacterial activity.

**Table 2:** Inhibition zone of the actinomycete isolates against the test pathogens.

Test pathogens									
S.No.	Isolates code	Gram-negative bacteria					Gram-positive bacteria		Fungi
		Ec	Kp	Pa	St1	St2	Sa	Se	*A. niger*
1	RVE002	32 ± 0.45	30 ± 0.20	27 ± 0.20	17 ± 0.58	20 ± 0.96	12 ± 0.35	20 ± 0.30	22 ± 0.58
2	RVE004	—	14 ± 0.31	14 ± 0.50	24 ± 0.16	28 ± 0.65	27 ± 0.77	21 ± 0.30	11 ± 0.6
3	RVE029	24 ± 0.45	—	—	13 ± 0.16	15 ± 0.96	25 ± 0.77	26 ± 0.30	14 ± 0.6
4	RVE077	17 ± 0.33	8 ± 0.20	27 ± 0.50	—	—	10 ± 0.50	13 ± 0.58	20 ± 0.0
5	RVE116	16 ± 0.20	22 ± 0.50	—	15 ± 0.60	—	16 ± 0.58	15 ± 1.16	12 ± 0.58
6	RVE118	15 ± 0.20	12 ± 0.50	16 ± 0.58	17 ± 0.60	17 ± 0.65	20 ± 1.00	20 ± 0.80	17 ± 1.20
7	RVE125	25 ± 0.33	5 ± 0.31		15 ± 0.96	27 ± 1.00	30 ± 0.60	30 ± 0.60	10 ± 0.00
8	RVE129	>35	22 ± 0.36	17 ± 0.40	>31	>31	>35	>40	30 ± 0.0
9	RVE133	22 ± 0.60	30 ± 0.31	25 ± 0.40	22 ± 0.58	28 ± 1.00	20 ± 1.00	10 ± 0.60	18 ± 0.58
10	RVE144	18 ± 0.45	—	–	26 ± 0.58	19 ± 1.00	21 ± 0.50	—	10 ± 0.88
11	RVE165	10 ± 0.60	—	25 ± 0.40	12 ± 0.58	12 ± 0.36	12 ± 0.50	—	12 ± 0.58
12	RVE172	20 ± 0.60	19 ± 0.31	32 ± 0.58	—	29 ± 0.50	12 ± 0.50	14 ± 0.40	—
13	RVE187	>31	27 ± 0.31	28 ± 0.10	>35	>35	>40	>40	27 ± 0.0
14	RVE193	15 ± 0.33	7 ± 0.50	5 ± 0.10	17 ± 0.60	21 ± 1.00	20 ± 0.35	22 ± 1.16	10 ± 0.58
15	RVE194	13 ± 0.33	18 ± 0.50	19 ± 0.58	15 ± 0.60	18 ± 1.00	16 ± 1.16	16 ± 1.16	17 ± 0.60
16	RVE214	15 ± 0.33	18 ± 0.36	20 ± 0.20	16 ± 0.80	15 ± 0.50	32 ± 0.35	33 ± 0.58	18 ± 1.20
17	RVE217	28 ± 0.20	18 ± 0.36	28 ± 0.20	>35	>35	>35	>35	30 ± 0.0
18	RVE219	18 ± 0.20	19 ± 0.50	18 ± 0.40	—	—	17 ± 0.58	14 ± 0.80	20 ± 0.6
19	RVE226	15 ± 0.45	22 ± 0.36	18 ± 0.58	15 ± 0.8	10 ± 0.65	33 ± 0.50	33 ± 0.58	—
20	RVE227	21 ± 0.20	16 ± 0.20	25 ± 0.40	22 ± 0.16	16 ± 0.36	17 ± 0.58	—	18 ± 0.6
21	RVE234	25 ± 0.33	15 ± 0.31	9 ± 0.10	31 ± 0.58	20 ± 0.50	11 ± 1.00	14 ± 0.30	—
22	RVE239	—	8 ± 0.36	8 ± 0.10	10 ± 0.58	14 ± 0.50	24 ± 0.77	34 ± 0.30	11 ± 0.58
23	RVE257	21 ± 0.45	—	—	16 ± 0.58	15 ± 1.00	26 ± 0.77	6 ± 0.30	14 ± 0.58
24	RVE258	19 ± 0.60	—	21 ± 0.20	30 ± 0.16	19 ± 0.36	15 ± 0.77	13 ± 0.58	20 ± 0.0

Note: values are mean of three replicates ± standard deviation (SD). (—): no antimicrobial activity; Sa: *Staphylococcus aureus*; Ec: *Escherichia coli*; Pa: *Pseudomonas aeruginosa*; Kp: *Klebsiella pneumoniae*; St1: *Salmonella typhi* ATCC-13311; St2: *Salmonella typhi* ATCC-14028; Se: *Staphylococcus epidermidis*; *A*. *niger*.

**Table 3:** The antimicrobial activity (mm) of the isolates against bacterial pathogens during secondary screening.

S.No.	Test pathogens								
	Isolates code	Gram-negative bacteria					Gram-positive bacteria		Fungi
		Ec	Kp	Pa	St1	St2	Sa	Se	*A. niger*
1	RVE002	19 ± 0.6^i^	12 ± 0.0^de^	16 ± 0.6^g^	15 ± 0.0^fg^	12 ± 0.6^ef^	22 ± 0.0^g^	19 ± 1.0^k^	22 ± 1.2^j^
2	RVE004	5 ± 0.0^b^	0 ± 0.0^a^	18 ± 1.2^f^	13 ± 1.2^de^	0 ± 0.0^a^	9 ± 1.0^cd^	8 ± 0.0^bc^	17 ± 0.6^h^
3	RVE029	0 ± 0.0^a^	10 ± 0.6^cd^	14 ± 0.0^c^	7 ± 0.0^b^	12 ± 0.6^ef^	17 ± 0.0^f^	24 ± 0.6^l^	16 ± 0.0^h^
4	RVE077	17 ± 0.0^h^	12 ± 0.6^de^	9 ± 0.0^b^	15 ± 0.6^fg^	18 ± 0.6^h^	14 ± 1.2^e^	0 ± 0.0^a^	9 ± 0.0^ef^
5	RVE116	15 ± 1.2^g^	14 ± 0.0^f^	16 ± 0.0^de^	6 ± 2. 3^b^	0 ± 0.0^a^	7 ± 0.0 ^c^	9 ± 0.0^cd^	8 ± 0.0^ef^
6	RVE118	7 ± 0.0^c^	9 ± 1.0^c^	14 ± 0.6^c^	11 ± 0.0^cd^	12 ± 0.6^ef^	17 ± 0.0 ^f^	17 ± 1.0^j^	5 ± 0.0^b^
7	RVE125	19 ± 0.0^i^	14 ± 0.0^f^	9 ± 0.6^b^	11 ± 0.0^cd^	9 ± 0.6^c^	21 ± 0.6^g^	14 ± 0.0^gh^	9 ± 0.0^ef^
8	RVE129	21 ± 0.6^jk^	25 ± 0.0^j^	20 ± 0.0^h^	18 ± 0.0^gh^	18 ± 0.0^h^	27 ± 0.0^i^	22 ± 0.0^l^	22 ± 0.6^j^
9	RVE133	0 ± 0.0^a^	17 ± 1.0^g^	20 ± 0.0^h^	17 ± 0.6^gh^	0 ± 0.0^a^	12 ± 1.2^cd^	10 ± 0.6^cd^	12 ± 0.6^g^
10	RVE144	0 ± 0.0^a^	10 ± 1.0^cd^	9 ± 0.0^b^	0 ± 0.0^a^	0 ± 0.0^a^	15 ± 1.0 ^f^	16 ± 1.0^h^	16 ± 0.3^h^
11	RVE165	12 ± 0.0^de^	17 ± 0.6^g^	16 ± 0.6^de^	11 ± 0.0^cd^	14 ± 0.6^fg^	11 ± 0.0^cd^	14 ± 0.0^g^	10 ± 1.2^g^
12	RVE172	0 ± 0.0^a^	10 ± 0.0^cd^	14 ± 0.6^c^	0 ± 0.0^a^	0 ± 0.0^a^	19 ± 0.0^g^	19 ± 1.0^j^	5 ± 0.0^b^
13	RVE187	25 ± 0.6^l^	27 ± 0.0^k^	22 ± 0.0^h^	20 ± 1.2^i^	21 ± 0.0^i^	25 ± 1.2^h^	25 ± 0.6^l^	16 ± 0.3^h^
14	RVE193	22 ± 1.2^k^	15 ± 1.0^fg^	18 ± 0.0^f^	8 ± 0.0^c^	9 ± 0.6^c^	11 ± 0.6^cd^	13 ± 0.0^f^	9 ± 0.6^ef^
15	RVE194	15 ± 1.2^g^	13 ± 0.0^f^	15 ± 1.2^cd^	20 ± 0.6^i^	21 ± 0.6^i^	12 ± 1.0^e^	19 ± 1.0^j^	17 ± 1.2^i^
16	RVE214	7 ± 0.6^c^	11 ± 0.6^de^	14 ± 0.6^c^	7 ± 0.6^b^	14 ± 0.0^fg^	5 ± 1.0^b^	11 ± 1.0^e^	17 ± 0.6^i^
17	RVE217	19 ± 0.0^i^	25 ± 0.6^j^	18 ± 0.6^f^	22 ± 1.2^j^	18 ± 0.0^h^	26 ± 0.6^i^	25 ± 0.0^l^	16 ± 0.0^h^
18	RVE219	13 ± 0.0^ef^	8 ± 0.6^c^	14 ± 1.0^c^	8 ± 2.3^c^	8 ± 0.6^b^	7 ± 0.6^b^	15 ± 0.6^h^	18 ± 0.0^i^
19	RVE226	13 ± 0.6^ef^	8 ± 1.0^c^	9 ± 1.2^b^	0 ± 0.0^a^	11 ± 0.6^cde^	5 ± 1.0^b^	0 ± 0.0^a^	11 ± 0.3^g^
20	RVE227	20 ± 0.0^jk^	22 ± 0.0^i^	18 ± 0.6^f^	15 ± 0.0^fg^	18 ± 0.6^h^	15 ± 1.2^f^	10 ± 0.6^cd^	5 ± 0.0^b^
21	RVE234	14 ± 0.6^g^	15 ± 0.0^fg^	17 ± 0.6^d^	18 ± 0.6^gh^	18 ± 0.0^h^	21 ± 0.6^g^	22 ± 0.0^k^	10 ± 0.0^fg^
22	RVE239	17 ± 0.0^h^	16 ± 1.0^g^	15 ± 0.0^cd^	20 ± 0.0^i^	14 ± 0.6^fg^	15 ± 0^f^	18 ± 0.0^i^	9 ± 0.0^ef^
23	RVE247	12 ± 0.6^de^	14 ± 0.6^f^	8 ± 0.0^b^	10 ± 0.0^c^	14 ± 0.6^fg^	15 ± 0.6^f^	16 ± 1.0^h^	11 ± 1.2^g^
24	RVE248	11 ± 1.2^cd^	14 ± 0.0^f^	12 ± 0.6^de^	10 ± 1.2^c^	9 ± 0.6^c^	17 ± 1.2^h^	15 ± 0.6^h^	16 ± 0.0^h^
	Control drug	28 ± 0.58^m^	25 ± 0.8^j^	24 ± 0.58^i^	29 ± 0.58^k^	28 ± 0.3^j^	25 ± 0.58^h^	25 ± 0.8^l^	29 ± 0.6^k^
	Negative control	0 ± 0.0^a^	0 ± 0.0^a^	0 ± 0.0^a^	0 ± 0.0^a^	0 ± 0.0^a^	0 ± 0.0^a^	0 ± 0.0^a^	0 ± 0.0^a^

Note: Sa: *Staphylococcus aureus*; Se: *Staphylococcus epidermidis*; *S*t1: *Salmonella typhi* ATCC-13311; Kp: *Klebsiella pneumonia*e; Ec: *Escherichia coli*; St2: *Salmonella typhi* ATCC-14028; Pa: *Pseudomonas aeruginosa*. Mean values sharing the same superscripts within a column are not significantly different at *p* < 0.05; inhibition zone diameters are expressed as mean ± SD of the three replications.

**Table 4:** Morphological, physiological, and biochemical characteristics of the test isolates.

Actinomycete isolates																								
Growth media	RVE 002	RVE004	RVE029	RVE077	RVE116	RVE118	RVE125	RVE129	RVE133	RVE144	RVE165	RVE172	RVE187	RVE193	RVE194	RVE214	RVE217	RVE219	RVE226	RVE227	RVE234	RVE239	RVE247	RVE248
ISP 2	+	++	+++	+++	++	+++	++	+++	++	++	+++	++	++	++	+++	++	+++	++	+++	+++	++	++	+++	+++
ISP 3	+++	+++	+++	++	++	+	+	++	++	+++	+	+++	+++	+++	+	+++	+++	+++	++	+++	++	+++	++	++
ISP 4	+++	+++	++	+++	+++	+++	++	++	+++	++	+++	+++	+++	+++	+++	++	+++	++	++	++	+++	+++	++	+++
ISP 5	++	+	++	++	+++	+	+	+	++	+	+	+	++	+	++	++	++	++	+	+	++	++	+++	++
ISP 6	+++	+++	+	+	+++	++	+++	+++	+++	++	++	++	+++	++	+++	+++	+++	+	++	++	+++	++	+	+++
ISP 7	++	++	+++	+++	++	+	++	+++	+	++	++	+	+++	++	+	+	+	++	++	++	++	++	+	+
SCA	++	+++	+++	++	++	+++	+++	+++	+++	+++	+	+	++	+++	+++	+++	+	++	++	+++	+++	++	++	++
Enzyme production																								
Urease	++	−	−	−	−	++	−	−	+	−	−	−	++	++	−	−	−	−	++	−	+	−	−	+
Amylase	+	+	+	+	+	+	+	+	+	+	+	+	+	+	+	+	+	+	+	+	+	+	+	+
Chitinase	+++	+	++	++	−	−	−	+++	−	−	+	+	+	+	+	+	+	+	+	+	+	+	+	+
Oxidase	+	−	−	+	+	+	++	++	++	−	++	++	+	+	++	+	−	+	++	+	−	++	−	+
Catalase	+	+	+	+	+	+	+	+	+	+	+	+	+	+	+	+	+	+	+	+	+	+	+	+
Caesinase	+	+	+	+	+	+	−	+	+	+	+	−	+	+	+	+	−	+	+	+	+	+	−	+
Nitrate reductase	−	−	++	−	−	++	−	++	−	++	++	−	++	−	−	−	++	−	−	−	−	−	++	++
Gelatin hydrolysis	+	−	+	+	−	+	+	+	+	+	+	−	+	−	+	+	−	+	+	−	+	−	+	−
Citrate utilization	+	+	−	+	+	−	+	+	−	+	+	+	+	+	+	−	−	+	+	+	+	−	+	+
Melanin formation	++	+	−	++	+++	+	+	+	++	+	+	++	+	++	++	+	++	++	+++	−	+	+	++	−
Optimum pH	7.5	7.5	7.5	7	8	8	7.5	7.5	8	7	7.5	7	8	7.5	7	7.5	7.5	7.5	7	7.5	8	7.5	8	7
Optimum NaCl	2.5	5	5	2.5	5	2.5	2.5	5	5	2.5	5	2.5	2.5	2.5	2.5	5	2.5	5	5	5	2.5	2.5	5	5
Optimum T°(°C)	25	30	30	30	30	25	30	30	30	30	25	30	30	25	25	30	30	30	30	30	30	25	25	30

−, No growth; +, poor growth; ++, moderate growth; +++, abundant growth.

## Data Availability

Further data related to this study can be made available upon request.
